# The impact of magnetic resonance imaging anxiety on treatment adherence

**DOI:** 10.3389/fpsyt.2025.1658226

**Published:** 2025-11-03

**Authors:** Lixing Lei, Xiongxiong Yang, Nian Liu, Lingling Tang, Xin He, Xiaohua Huang

**Affiliations:** ^1^ Department of Radiology, Affiliated Hospital of North Sichuan Medical College, Nanchong, China; ^2^ Department of Radiology, Nanchong Hospital of Traditional Chinese Medicine, Nanchong, China

**Keywords:** magnetic resonance imaging, anxiety, treatment adherence, loss aversion, information avoidance

## Abstract

**Background:**

Magnetic resonance imaging (MRI) exams often induce higher levels of patient anxiety compared to other routine examinations due to factors such as enclosed spaces, prolonged immobility, and noise interference. This anxiety may interfere with cognitive function and reduce patients’ willingness to adhere to medical instructions. However, previous research has seldom investigated the impact of MRI anxiety on patients’ treatment adherence.

**Method:**

To address this gap, the current study employs a convenience sampling method to select 495 patients who underwent MRI examinations at a tertiary hospital in Sichuan Province between April and June 2025. We utilize the MRI Anxiety Scale, Loss Aversion Scale, Information Avoidance Scale, and Treatment Adherence Scale to conduct our investigation. Based on the Information asymmetry theory, this study constructs a structural equation model to analyze the relationship between MRI anxiety and treatment adherence, as well as the underlying mechanisms.

**Result:**

The results show that MRI anxiety is significantly negatively correlated with treatment adherence and significantly positively correlated with both loss aversion and information avoidance. Loss aversion and information avoidance partially mediate the association between MRI anxiety and treatment adherence. The findings suggest that higher levels of MRI anxiety are associated with lower treatment adherence, with loss aversion and information avoidance behaviors potentially contributing to this relationship.

**Conclusion:**

Therefore, clinical practice should include targeted interventions addressing loss aversion and information avoidance, such as optimizing risk communication strategies and providing structured informational support, to alleviate anxiety and improve treatment adherence, thereby providing a theoretical foundation and practical pathway to enhance MRI examination outcomes.

## 1 Introduction

Magnetic Resonance Imaging (MRI) is an imaging technique that reconstructs signals generated by the resonance of atomic nuclei in a magnetic field to produce images of the body’s internal structures and abnormalities (1, 2). In recent years, the psychological anxiety induced by MRI examinations, characterized by enclosed environments, loud noises, and prolonged examination times, has garnered significant attention from scholars (3, 4). For example, Oktay et al. (5) found that audiovisual interventions can effectively reduce MRI anxiety and improve image quality. Previous studies have focused on interventions to alleviate MRI anxiety to reduce patients’ psychological discomfort and pain and enhance image quality (6–8). However, despite these contributions, the impact of MRI anxiety on patients’ adherence to treatment has not received adequate attention. This oversight is unfortunate, as this influence may extend throughout the examination and subsequent treatment phases. Specifically, pre-examination anxiety may intensify patients’ perceived threat of the MRI environment, leading to behavioral avoidance such as hesitation, repeated reassurance-seeking, or requests to postpone the examination. These behaviors represent an attempt to regain a sense of control under uncertainty, yet paradoxically amplify cognitive and emotional distress, increasing the likelihood of examination refusal or incomplete cooperation (9). Some patients may even experience physical reactions due to anxiety, making it difficult to complete the examination, leading to interruptions or delays (10). During the examination, anxiety can distract patients’ attention, making it harder for them to remain still or follow instructions, potentially affecting image quality and necessitating repeated scans, which further increases patients’ psychological and financial burdens (11). Post-examination anxiety may manifest as excessive concern about the results, leading patients to repeatedly search for information online or through non-professional channels, entering a vicious cycle of information overload, misinterpretation, and escalating anxiety. In extreme cases, fear of being diagnosed with a severe condition may cause patients to avoid receiving reports or evade communication with physicians about the results. Particularly in the management of chronic diseases, cancer treatment, and neurological disorders, where the frequency and importance of MRI examinations are increasing (12), investigating the relationship between MRI anxiety and treatment adherence becomes even more critical.

Treatment adherence refers to the extent to which patients follow medical instructions for treatment and management (13, 14). Poor adherence can lead to adverse consequences, such as disease progression, increased cardiovascular risk, worsening health conditions, and hindered social integration (15, 16). Numerous studies have proposed targeted interventions from various perspectives, including psychological education, pharmacological interventions, mobile health applications, telemedicine, wearable devices (17, 18). For example, Butler et al. (19) found that using digital health technologies can effectively enhance patients’ treatment adherence. However, the impact of anxiety caused by medical devices and examinations on treatment adherence, particularly in the context of MRI, has not been adequately explored. In the MRI context, treatment adherence encompasses not only whether patients can successfully complete the MRI examination but also their subsequent adherence to treatment and follow-up based on the examination results. Patients’ anxiety may lead to refusal to cooperate with the examination, potentially resulting in unclear or distorted images that affect diagnostic accuracy (9). Additionally, for minor, early-stage, or special types of lesions, accurate detection of their size and location through MRI requires high patient adherence (20, 21). Despite the close relationship between MRI anxiety and treatment adherence, previous research has rarely analyzed or discussed this connection. Therefore, this study attempts to address the following questions: (1) Does MRI anxiety have a negative impact on patients’ treatment adherence? (2) What internal mechanisms mediate the relationship between MRI anxiety and treatment adherence?

Loss aversion theory provides a crucial framework for understanding MRI anxiety and its behavioral consequences. Loss aversion refers to the phenomenon where individuals are more sensitive to losses than to gains of the same magnitude (22, 23). This psychological tendency is particularly pronounced in medical decision-making, especially in situations involving invasive procedures or treatments. In other words, in medical contexts, patients tend to focus more on potential negative outcomes than on potential benefits (24), a tendency that significantly impacts decision quality and treatment adherence. For example, Reitich-Stolero et al. (25) noted that loss aversion in medical decision-making often stems from individuals’ heightened sensitivity to unknown risks and short-term discomfort. Therefore, in the context of imaging examinations, patients often overemphasize the discomfort or potential risks associated with the procedure, leading to excessive anxiety or irrational refusal of necessary examinations (26). In essence, MRI anxiety can be viewed as a fear of potential “losses,” such as losses in health, safety, or control. When patients undergo MRI examinations, they may associate the process with the potential revelation of unfavorable diagnoses, leading to negative expectations about their future health. This heightened sensitivity to loss directly influences patients’ psychological states and behavioral choices, thereby affecting their adherence to treatment protocols.

Information avoidance is the tendency for individuals to actively avoid exposure to information related to potential threats or negative outcomes (27, 28). While information avoidance may alleviate short-term anxiety, its long-term effects can negatively impact health behaviors (29). Recent research in the field of psychological health has revealed the complex role of information avoidance in health-related decision-making (30, 31). Studies have shown that information avoidance is significantly positively correlated with psychological distress and can indirectly lower individuals’ sense of control over health threats by delaying information processing and weakening problem-solving abilities, thereby influencing health behavior choices and execution (32–34). In the context of MRI examinations, the uncertainty of the examination process and the potential for painful or uncomfortable experiences, coupled with the possibility of revealing health issues, make patients more prone to information avoidance, thereby inducing anxiety. According to Emotion Regulation Theory, individuals consciously or unconsciously modify the onset, intensity, and expression of their emotions to maintain psychological equilibrium (35, 36). In the MRI context, anxiety activates regulatory mechanisms such as avoidance, enabling individuals to temporarily reduce emotional discomfort by minimizing exposure to threatening information. However, this behavior can lead to misunderstandings or underestimation of examination results, diminishing patients’ trust in diagnostic findings and their acceptance of treatment recommendations (37). Furthermore, information avoidance may exacerbate MRI anxiety, thereby negatively impacting subsequent treatment adherence. Additionally, information avoidance may weaken patients’ sense of control over their condition (38), indirectly influencing their cognitive evaluation of and behavioral willingness to adhere to treatment recommendations.

According to information asymmetry theory, asymmetric distribution of information in decision-making processes leads to systematic biases in choice, often stemming from misjudgments of risk and selective use of information (39, 40). In the context of MRI examinations, information asymmetry primarily manifests in patients’ uncertainty about the examination process, result interpretation, and subsequent treatment options. Based on this, patients’ uncertainty about the MRI process and its outcomes can activate loss aversion psychology, influencing their information processing patterns and making them overly sensitive to potential negative outcomes (41). This heightened sensitivity generates strong psychological discomfort, prompting patients to adopt information avoidance strategies (42), such as selectively avoiding information related to the examination or refraining from deeper understanding of examination details, to temporarily alleviate anxiety. However, information avoidance is not merely a passive behavior but rather an active process of selectively filtering information sources or avoiding in-depth exploration of examination details. While this behavior may reduce short-term anxiety, it can lead to long-term distortions in patients’ understanding of the examination’s purpose, process, and outcomes, thereby influencing their cognitive evaluation of and behavioral willingness to adhere to treatment recommendations.

Based on the above analysis, this study proposes the following hypotheses:

H1: MRI anxiety is negatively associated with patients’ treatment adherence.
H2: Loss aversion significantly mediates the association between MRI anxiety and treatment adherence.
H3: Information avoidance significantly mediates the association between MRI anxiety and treatment adherence.
H4: Loss aversion and information avoidance exhibit a significant chain mediation in the association between MRI anxiety and treatment adherence.

## 2 Methods

### 2.1 Participants

To validate the four hypotheses proposed, this study employed a convenience sampling method to collect data in the Radiology Department of a tertiary Grade A hospital in Sichuan Province. Participants were selected based on their availability and willingness to participate during their scheduled MRI examinations, as this approach was feasible given the clinical setting and time constraints (43). This non-probability sampling method was chosen to ensure adequate sample size and diversity within the study period (April to June 2025), though it may limit the generalizability of findings to broader populations. Prior to data collection, all measurement scales were developed into both electronic and paper questionnaires. These were distributed to professional clinical doctors and the Chief of the Radiology Department for evaluation and revision. Subsequently, the research team communicated the purpose, risks, and benefits of the study to the Chief of the Radiology Department. Upon obtaining consent, data collection commenced.

Sample size calculation. The minimum sample size was calculated using G*power 3.1 software (44). This study selected an F-test with a significance level (α) of 0.05, a desired power (1-β) of 0.8, and an effect size (f²) of 0.15. The results indicated that a minimum of 119 participants was required.

Data collection process: Patients who could use mobile electronic devices completed the electronic questionnaire, while those who could not use the paper-based version. During this process, each patient was informed about the risks, benefits, and procedures of the study to ensure their right to informed consent. After obtaining the patients’ signed consent forms, they proceeded to complete the questionnaires. Based on this process, a total of 513 patients were recruited for this survey, meeting the minimum sample size requirement.

Exclusion criteria: (1) patients who refused to sign the informed consent; (2) patients with severe language, cognitive or psychiatric impairment, unable to understand study instructions or independently complete questionnaires; (3) patients with severe mental disorders such as schizophrenia or severe bipolar disorder confirmed by psychiatric history or clinical evaluation; (4) patients who need MRI examination without physician confirmation; and (5) Patients who completed the questionnaire within 6 minutes were excluded from the responses with strong consistency. Severe impairment was defined as significant impairment in communication or cognitive function, such as the inability to respond coherently in patients with advanced dementia or severe aphasia (45, 46). Using Mini-Mental State Examination (MMSE), the patient’s score was less than 10 (47, 48). During the preexamination consultation, a brief cognitive screening using the MMSE was performed by a radiologist, supplemented by a review of medical records, for assessment. Patients with MMSE scores of 19-23 (indicating mild cognitive impairment) (49, 50) were included if they could provide informed consent and complete the questionnaire without assistance, as this enhances representativeness of the target population undergoing MRI. Patients with MMSE scores ≥24 were considered cognitively normal (51, 52). Given the potential variability in response accuracy due to mild cognitive or communication impairments, we excluded patients with communication difficulties from the study. Patients with moderate cognitive impairment (MMSE score of 11 to 18) were assigned a value of 1, those with mild cognitive impairment (MMSE score of 19 to 23) were assigned a value of 2, and those with normal cognitive function (MMSE score of 24 to 30) were assigned a value of 3; this category of patients was applied to subsequent analyses.

**Figure 1:**
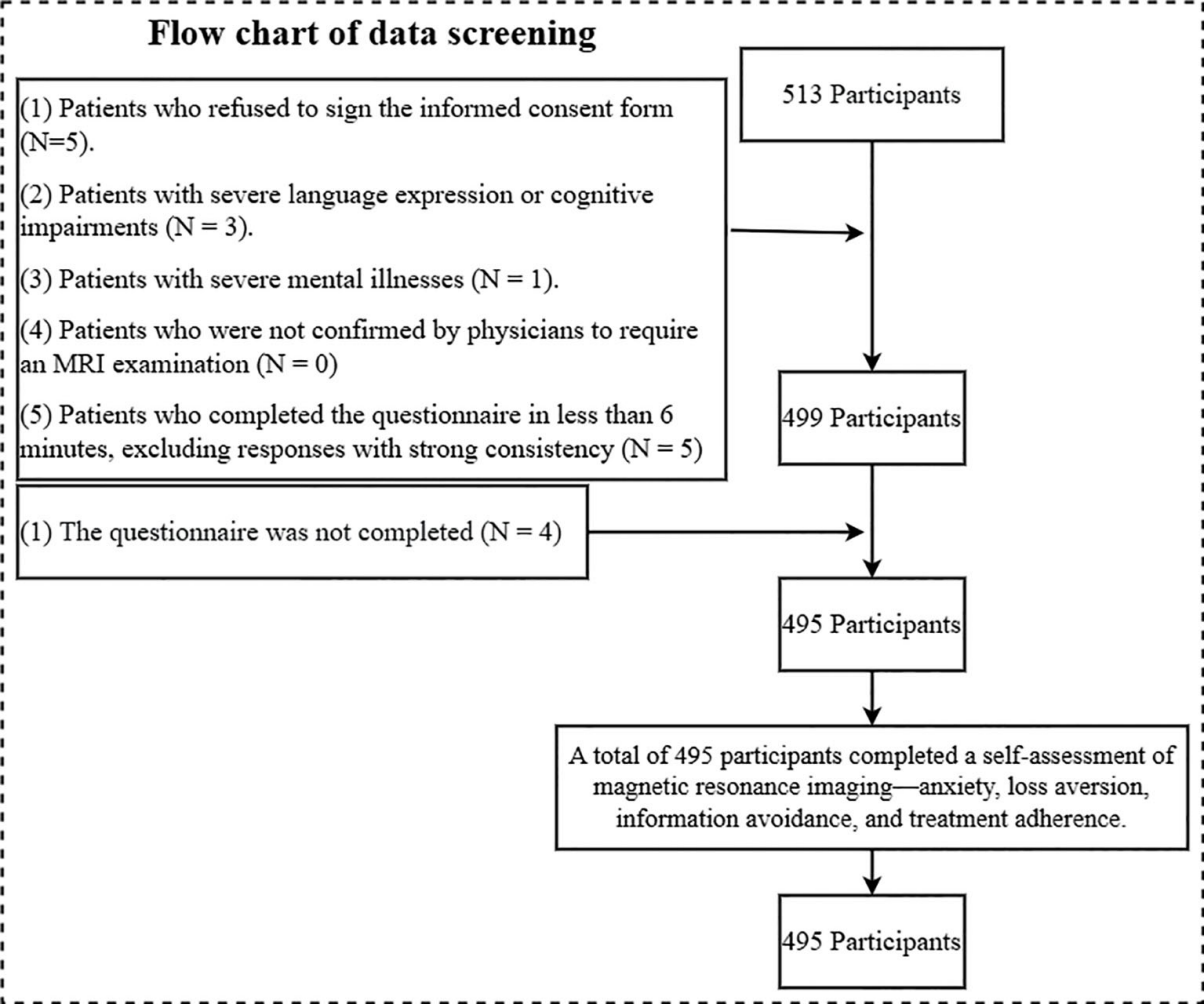
Data cleaning process.

Based on the above exclusion criteria, 18 participants were excluded during the data cleaning phase, with the specific exclusion process and numbers detailed in **Figure 1**. The final sample size was 495, with an effective response rate of 96.49%. Among these, there were 316 male patients and 179 female patients. Detailed demographic information is presented in **Table 1**.

**Table 1:** Demographic information for all participants.

Variables	Items	N	Percentage (%)
Gender	Male	316	63.8%
	Female	179	36.2%
Age	18–30 years old	91	18.4%
	31–45 years old	316	63.8%
	46–60 years old	79	16%
	Over 61 years of age	9	1.8%
Education	High school and below	312	63%
	Undergraduate college	121	24.4%
	Graduate student or above	62	12.5%
Place of residence	Cities	313	63.2%
	Countryside	182	36.8%
Marriage	Divorced	85	17.2%
	Widowed	12	2.4%
	Unmarried	229	46.3%
	Married	169	34.1%
Monthly income	≤ 3000 ¥	57	11.5%
	3001-6000 ¥	247	49.9%
	6001-9000 ¥	166	33.5%
	Over 9001 ¥	25	5.1%
Check the cause	Abdominal and pelvic diseases (e.g., pancreatitis, liver tumors, prostate)	51	10.3%
	Musculoskeletal disorders (e.g., fractures, arthritis, lumbar disc herniation)	216	43.6%
	Neurological disorders (e.g., cerebral infarction, brain tumors, epilepsy)	53	10.7%
	Cardiovascular diseases (e.g., heart disease, cardiac vascular malformations)	160	32.3%
	Cancer screening or follow-up (e.g., malignancy analysis, metastases, postoperative follow-up)	15	3%
Location	Abdomen	61	12.3%
	Spine/spinal cord	158	31.9%
	Pelvic cavity	19	3.8%
	Joints of the extremities	30	6.1%
	Head/brain	62	12.5%
	Chest	165	33.3%
Frequency of inspection	The first examination	134	27.1%
	2–3 times	204	41.2%
	4–5 times	140	28.3%
	More than 5 times	17	3.4%

### 2.2 Research tools

#### 2.2.1 Magnetic resonance imaging - anxiety scale

The Magnetic Resonance Imaging Anxiety Scale, developed by Ahlander et al. (53), consists of 15 measurement items designed to assess patients’ anxiety related to magnetic resonance imaging. Each item uses a 5-point Likert scale, ranging from 1 (“strongly disagree”) to 5 (“strongly agree”). Higher scores indicate greater anxiety levels regarding MRI. This scale has been widely applied to Chinese patient populations, demonstrating good cultural adaptability (54). In this study, the Cronbach’s α value for this scale was 0.904. Confirmatory factor analysis indicated good structural validity (CMIN/DF = 3.304, GFI = 0.917, AGFI = 0.890, RMSEA = 0.068, CFI = 0.924, TLI = 0.912).

#### 2.2.2 Loss aversion scale

The Loss Aversion Scale was adapted from Lam and Lin (55), originally developed to assess loss aversion in the context of cancer fear and screening behaviors. In the original scale, items focused on “cancer” as the potential loss domain; for this study, “cancer” was replaced with “potential negative outcomes from the MRI examination” to align with the MRI-specific context, capturing patients’ sensitivity to perceived losses such as health deterioration, loss of control, or disruption to daily life revealed by MRI results. This adaptation maintains the scale’s focus on how individuals weigh potential losses more heavily than equivalent gains, manifesting as reluctance to engage with threatening scenarios to preserve current psychological gains (e.g., hope or normalcy).

The scale consists of 4 items, each rated on a 5-point Likert scale ranging from 1 (“strongly disagree”) to 5 (“strongly agree”), with higher scores indicating greater loss aversion. The adapted items are: (1) I would rather not think about potential negative outcomes from the MRI examination because not knowing helps me maintain hope; (2) I would rather not think about potential negative outcomes from the MRI examination and enjoy the life that I have; (3) Being constantly reminded of potential negative outcomes from the MRI examination makes me nervous about my own health condition; (4) Being diagnosed with a condition based on the MRI examination means I would have to stop all activities to seek treatment.

These items relate to the construct of loss aversion, as defined in the introduction (individuals are more sensitive to losses than to gains of the same magnitude) (22, 23), by operationalizing it in health decision-making contexts. Rooted in prospect theory Kai-Ineman and Tversky (56), loss aversion leads to status quo bias, where individuals avoid actions or thoughts that risk losses (e.g., confronting bad MRI results, which could “lose” hope or lifestyle) despite potential gains (e.g., early intervention). Unlike traditional behavioral paradigms using gambles (e.g., preferring no change over a 50% chance of gain/loss to reveal aversion), this self-report scale captures cognitive manifestations in real-world health scenarios, where loss aversion influences avoidance of diagnostic processes to prevent perceived asymmetrical losses. This adaptation is theoretically justified in health psychology, as loss aversion in medical contexts often amplifies fear of negative outcomes, reducing adherence (Tamar 25).

Importantly, while some items involve “not thinking about” threats, which may superficially resemble information avoidance (active dodging of info to reduce distress), the constructs are distinct: loss aversion reflects an emotional bias in valuing losses over gains, leading to broader decision biases, whereas information avoidance is a specific cognitive strategy. The scale’s mediation role in the SEM (distinct from information avoidance paths) empirically supports this separation.

In this study, the Cronbach’s α value for the adapted Loss Aversion Scale was 0.760, indicating acceptable internal consistency. Confirmatory factor analysis supported good structural validity (CMIN/DF = 2.193, GFI = 0.996, AGFI = 0.979, RMSEA = 0.049, CFI = 0.995, TLI = 0.985), confirming the scale’s suitability for assessing loss aversion in the MRI context.

#### 2.2.3 Information avoidance scale

The Information Avoidance Scale was developed and validated by Howell and Shepperd (57). The scale consists of 8 measurement items specifically designed to assess information avoidance related to MRI examinations. This scale has been widely applied to Chinese patient populations, for example, in Gu et al. (58), where it was used to analyze health information avoidance among patients undergoing mindfulness interventions. Thus, it demonstrated good cultural adaptability and was deemed appropriate for assessing information avoidance in MRI contexts. In this study, blanks were filled with “the results of the MRI examination” to focus on MRI-specific avoidance, without referencing participants’ exact diseases (e.g., cancer, brain damage) for generalizability across diverse conditions (**Table 1**). Example items: (1) “I would rather not know the results of the MRI examination”; (2) “I would avoid learning the results of the MRI examination”; (3) “Even if it will upset me, I want to know the results of the MRI examination” (reverse coded); (4) “When it comes to the results of the MRI examination, sometimes ignorance is bliss”; (5) “I want to know the results of the MRI examination” (reverse coded); (6) “I can think of situations in which I would rather not know the results of the MRI examination”; (7) “It is important to know the results of the MRI examination” (reverse coded); (8) “I want to know the results of the MRI examination immediately” (reverse coded). Each item uses a 5-point Likert scale (1 = “strongly disagree,” 5 = “strongly agree”). Higher scores indicate a stronger tendency to avoid information. In this study, the Cronbach’s α value for this scale was 0.870. Confirmatory factor analysis indicated good structural validity (CMIN/DF = 1.772, GFI = 0.983, AGFI = 0.970, RMSEA = 0.040, CFI = 0.989, TLI = 0.985).

#### 2.2.4 Treatment adherence scale

The Treatment Adherence Scale was adapted from the Chinese version of the Morisky Medication Adherence Scale-8, as validated by Zhao et al. (59) for assessing warfarin adherence in Chinese patients. The MMAS-8 is originally based on Morisky et al. (60), a foundational tool for measuring self-reported adherence, with strong psychometric properties (original internal consistency α = 0.83, predictive validity for clinical outcomes). Although Morisky et al. (61), which further validated the scale, was retracted in 2023 due to intellectual property disputes, the core MMAS-8 items remain widely used and psychometrically sound in adapted forms, as in Wang et al. (62). In this study, the scale was modified to evaluate patients’ adherence to MRI-related treatment instructions, including cooperation during the examination and follow-up based on results, rather than medication-specific adherence. Each item uses a 5-point Likert scale (1 = “strongly disagree,” 5 = “strongly agree”). Higher scores indicate greater treatment adherence. The adapted items are as follows: (1) Do you sometimes forget to follow the MRI-related treatment instructions?; (2) Thinking over the past 2 weeks, were there any days when you forgot to follow the MRI-related treatment instructions?; (3) Have you ever cut back or stopped following the MRI-related treatment instructions because you felt worse when you followed them?; (4) When you travel or leave home, do you sometimes forget to follow the MRI-related treatment instructions?; (5) Didn’t you follow the MRI-related treatment instructions yesterday?; (6) When you feel better, do you sometimes stop following the MRI-related treatment instructions?; (7) Do you ever feel hassled about sticking to the MRI-related treatment regimen?; (8) How often do you have difficulty remembering to follow the MRI-related treatment instructions?.

In this study, the Cronbach’s α value was 0.847, indicating high reliability. Confirmatory factor analysis demonstrated good structural validity (CMIN/DF = 1.906, GFI = 0.985, AGFI = 0.971, RMSEA = 0.043, CFI = 0.988, TLI = 0.983). This adaptation ensures relevance to the MRI context while maintaining the scale’s established validity.

## 3 Result

### 3.1 Normality test

Following Kline (63) study, we considered that when the absolute skewness of the data samples is less than 3 and the kurtosis is less than 8, the data are approximately normally distributed. In this study, descriptive analysis was used to test the skewness and kurtosis of each variable. The data results showed that both the skewness and kurtosis of all variables met the requirements, as shown in **Table 2**. Therefore, the data samples in this study conformed to a normal distribution.

**Table 2:** Normality analysis of each variable.

Variables	M	SD	Skewness	Kurtosis
Magnetic Resonance Imaging - Anxiety	3.142	0.694	-0.072	0.487
Loss Aversion	3.096	0.846	-0.064	-0.137
Information Avoidance	3.094	0.813	-0.083	-0.009
Treatment Adherence	3.175	0.791	-0.144	0.306

### 3.2 Common method bias test

Common method bias arises due to single-source bias, same-context measurement bias, or similarity in measurement formats, leading to artificial covariance between predictor and criterion variables (64). To mitigate this, we informed the participants that their data would be strictly confidential and anonymous at the data collection stage to reduce social desirability bias and subsequently minimize common method bias (65).

Additionally, we conducted Harman’s single-factor test by including all measurement items in an exploratory factor analysis. The results indicated that the study extracted 4 factors with eigenvalues greater than 1, and the variance explained by the first factor was below the critical threshold of 50%. Therefore, no common method bias was found in this study.

### 3.3 One-way ANOVA of demographic information and core variables

Referring to the study of Gui et al. (66), in order to further enhance the accuracy of the experimental results, this study used all the demographic information of the patients as independent variables and the core variables as dependent variables, using one-way ANOVA. The results showed that the gender of patients had a significant effect on loss aversion. The age of the patient had a significant effect on loss aversion. Differences in education, marriage, and place of residence of the patients did not significantly affect the core variables. The patient’s income level had a significant effect on MRI anxiety, loss aversion, and information avoidance. There was no significant difference in any of the core variables by patient reason for testing. Patient examination site showed significant differences for all core variables. Loss aversion, information avoidance, and treatment adherence were significantly affected by the number of examinations performed by patients. The type of cognitive function of patients has a significant impact on MRI anxiety and information avoidance. The specific one-way ANOVA results are shown in **Table 3**.

**Table 3:** Summary table of one-way ANOVA results.

Variables	Items	MRI anxiety				Loss aversion				Information avoidance				Treatment adherence			
		M	SD	F	P	M	SD	F	P	M	SD	F	P	M	SD	F	P
Gender	Male	3.122	0.697	0.734	0.392	3.021	0.858	7.029	0.008	3.05	0.811	2.565	0.11	3.146	0.782	1.176	0.279
	Female	3.178	0.689			3.229	0.808			3.172	0.812			3.227	0.805		
Age	18–30 years old	3.111	0.770	0.977	0.403	3.077	0.898	3.378	0.018	3.059	0.919	0.727	0.536	3.160	0.827	0.543	0.653
	31–45 years old	3.121	0.698			3.033	0.852			3.085	0.803			3.153	0.802		
	46–60 years old	3.241	0.578			3.313	0.716			3.131	0.719			3.275	0.691		
	Over 61 years of age	3.356	0.639			3.583	0.800			3.458	0.824			3.254	0.883		
Education	Undergraduate college	3.143	0.678	0.054	0.948	3.069	0.825	1.034	0.356	3.067	0.804	0.482	0.618	3.204	0.789	0.558	0.573
	High school and below	3.131	0.804			3.093	0.925			3.131	0.866			3.119	0.831		
	Graduate student or above	3.167	0.529			3.238	0.782			3.157	0.752			3.143	0.716		
Marriage	Divorced	3.056	0.574	0.704	0.550	2.974	0.716	1.046	0.372	3.037	0.678	0.656	0.580	3.119	0.751	0.225	0.879
	Widowed	3.278	0.403			3.313	0.740			3.240	0.526			3.119	0.568		
	Unmarried	3.146	0.649			3.094	0.845			3.139	0.800			3.180	0.753		
	Married	3.173	0.814			3.145	0.910			3.052	0.904			3.201	0.873		
Place of residence	Cities	3.149	0.737	0.063	0.801	3.124	0.879	0.923	0.337	3.075	0.872	0.490	0.484	3.193	0.845	0.4	0.528
	Countryside	3.133	0.613			3.048	0.785			3.128	0.700			3.146	0.688		
Monthly income	level ≤3000 ¥	2.827	0.781	7.225	<0.001	2.803	1.009	4.168	0.006	2.693	0.921	5.844	<0.001	3.003	0.817	2.388	0.068
	3001-6000¥	3.137	0.645			3.134	0.787			3.129	0.748			3.158	0.755		
	6001-9000¥	3.202	0.627			3.084	0.781			3.146	0.762			3.214	0.767		
	Over 9001¥	3.531	1.043			3.470	1.197			3.325	1.186			3.486	1.108		
Check the cause	Abdominal and pelvic diseases (e.g., pancreatitis, liver tumors, prostate)	3.149	0.558	1.665	0.157	3.167	0.817	1.585	0.177	3.184	0.768	1.003	0.406	3.196	0.797	1.665	0.157
	Musculoskeletal disorders (e.g., fractures, arthritis, lumbar disc herniation)	3.165	0.690			3.097	0.819			3.064	0.804			3.213	0.757		
	Neurological disorders (e.g., cerebral infarction, brain tumors, epilepsy)	2.914	0.903			2.835	1.083			2.936	1.053			2.925	0.989		
	Cardiovascular diseases (e.g., heart disease, cardiac vascular malformations)	3.179	0.645			3.159	0.770			3.161	0.720			3.213	0.723		
	Cancer screening or follow-up (e.g., malignancy analysis, metastases, postoperative follow-up)	3.227	0.748			3.083	1.072			3.067	1.027			3.048	1.051		
Location	Abdomen	3.421	0.648	4.159	0.001	3.447	0.740	4.858	<0.001	3.410	0.734	5.730	<0.001	3.466	0.723	4.31	<0.001
	Spine/spinal cord	3.159	0.692			3.090	0.851			3.116	0.793			3.192	0.754		
	Pelvic cavity	3.193	0.692			3.158	0.791			3.092	0.746			3.060	0.872		
	Joints of the extremities	3.142	0.732			3.067	0.871			2.729	0.857			3.190	0.846		
	Head/brain	2.863	0.846			2.714	1.036			2.750	0.966			2.827	0.916		
	Chest	3.124	0.603			3.114	0.746			3.152	0.736			3.194	0.738		
Frequency of inspection	2–3 times	3.152	0.686	1.928	0.124	3.158	0.840	2.994	0.031	3.153	0.803	5.507	<0.001	3.195	0.803	4.044	0.007
	4–5 times	3.212	0.532			3.182	0.713			3.221	0.677			3.309	0.651		
	More than 5 times	3.318	0.931			3.088	1.053			3.206	1.031			3.303	0.913		
	The first examination	3.035	0.805			2.912	0.931			2.858	0.884			2.990	0.859		
MMSE	Moderate cognitive impairment	3.180	0.714	3.192	0.042	3.233	0.885	1.364	0.256	3.221	0.767	3.373	0.035	2.797	0.744	1.634	0.196
	Mild cognitive impairment	3.302	0.701			3.181	0.817			3.263	0.778			2.694	0.787		
	Normal cognitive function	3.099	0.685			3.059	0.847			3.037	0.821			2.860	0.795		

### 3.4 Correlation analysis

We conducted a correlation analysis of MRI anxiety, loss aversion, information avoidance, and treatment adherence, as shown in **Table 4**. The results showed: MRI anxiety was significantly negatively correlated with treatment adherence (r = -0.732, P < 0.001); MRI anxiety was significantly positively correlated with loss aversion (r = 0.753, P < 0.001); MRI anxiety was significantly positively correlated with information avoidance (r = 0.687, P < 0.001); loss aversion was significantly positively correlated with information avoidance (r = 0.744, P < 0.001); loss aversion was significantly negatively correlated with treatment adherence (r = -0.714, P < 0.001); and information avoidance was significantly negatively correlated with treatment adherence (r = -0.753, P < 0.001). These results provided the conditions for further mediation testing.

**Table 4:** The results of correlation analysis were summarized.

Variables	1	2	3	4
1. MRI Anxiety	1			
2. Loss Aversion	0.753***	1		
3. Information Avoidance	0.687***	0.744***	1	
4. Treatment Adherence	-0.732***	-0.714***	-0.753***	1

***p < 0.001.

Based on SPSS guidance from Field (67) and American Psychological (68), the number of comparisons in this study (K = n (n-1)/2) with an initial alpha level of 0.05 and Bonferroni corrected alpha level (α_bonF = α/k). Correlation comparison results: P-value ≤ α_bonF, after Bonferroni correction, the correlation was statistically significant at the α=0.05 level; However, the p-value was greater than α_bonF, and the correlation was no longer statistically significant at the α=0.05 level after Bonferroni correction.

The results show that the number of comparisons (K) is 6, and the value of α_bonf is 0.0083. The P values of the correlation coefficients according to **Table 4** are all less than α_bonF. After this adjustment, all reported associations remained statistically significant, as the unadjusted p values were all < 0.001, well below the adjusted threshold. This conservative adjustment confirmed the robustness of the association between MRI anxiety, loss aversion, information avoidance, and treatment adherence.

### 3.5 Mediation analysis of loss aversion

**Figure 2:**
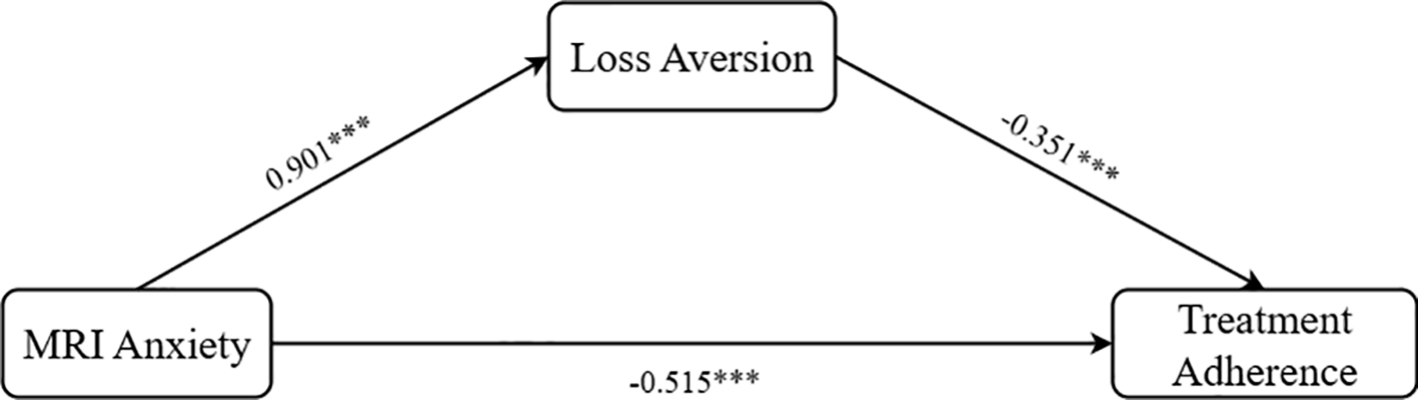
Path coefficient plot of the mediating effect of loss aversion. ***p < 0.001.

After controlling for factors such as gender, age, monthly income, frequency of inspection, check the cause and types of cognitive functions (MMSE score), we used loss aversion as the mediator, MRI anxiety as the independent variable, and treatment adherence as the dependent variable. The Process Model 4 was employed to test the mediating effect of loss aversion (Bootstrap sample: 5000; Igartua and Hayes (69)), validating Hypothesis H2. The results demonstrated: MRI anxiety was significantly positively associated with loss aversion (β = 0.901, P < 0.001, 95% CI = [0.838, 0.982]); MRI anxiety was significantly negatively associated with treatment adherence (β = -0.515, P < 0.001, 95% CI = [-0.614, -0.415]); and loss aversion was significantly negatively associated with treatment adherence (β = -0.351, P < 0.001, 95% CI = [-0.432, -0.269]). Overall, loss aversion exhibited a significant partial mediating effect between MRI anxiety and treatment adherence (β = -0.319, SE = 0.049, 95% CI = [-0.416, -0.221]), as shown in **Figure 2**, thereby validating Hypothesis H2.

### 3.6 Mediation analysis of information avoidance

**Figure 3:**
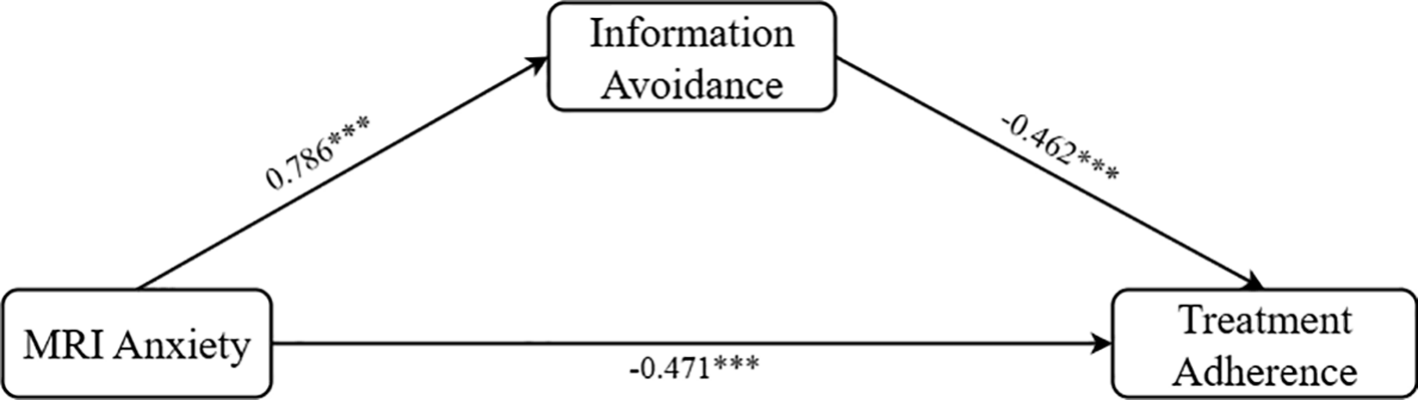
Path coefficient plot of the mediating effect of information avoidance. ***p < 0.001.

Subsequently, we retained the same control variables and used information avoidance as the mediator. The Process Model 4 was again applied to test the mediating effect (Bootstrap sample: 5000; Igartua and Hayes (69)), this time verifying Hypothesis H3. The results indicated: MRI anxiety had a significant associated on information avoidance (β = 0.786, P < 0.001, 95% CI = [0.709, 0.863]); MRI anxiety had a significant associated on treatment adherence (β = -0.471, P < 0.001, 95% CI = [-0.554, -0.388]); and information avoidance had a significant associated on treatment adherence (β = -0.462, P < 0.001, 95% CI = [-0.533, -0.391]). Overall, information avoidance displayed a significant partial mediating effect between MRI anxiety and treatment adherence (β = -0.363, SE = 0.046, 95% CI = [-0.457, -0.276]), as shown in **Figure 3**, thus validating Hypothesis H3.

### 3.7 Chain mediation analysis of loss aversion and information avoidance

**Figure 4:**
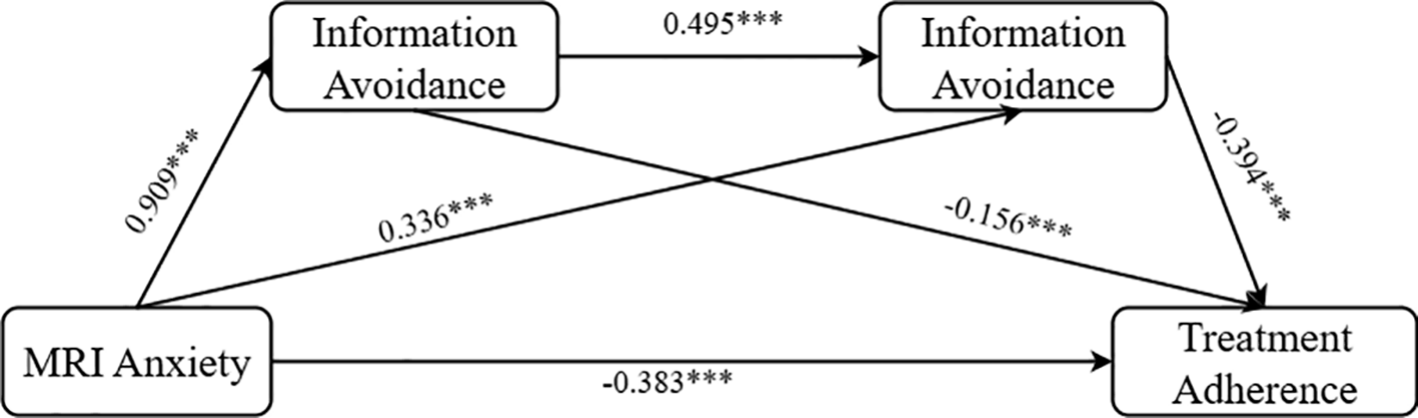
Graphs of chain mediation path coefficients for loss aversion and information avoidance. ***p < 0.001.

Finally, we treated both loss aversion and information avoidance as mediators, retained the same control variables, and utilized Process Model 6 to test their chain mediating effects (Bootstrap sample: 5000; Igartua and Hayes (69)). The results revealed: loss aversion had a significant associated on information avoidance (β = 0.495, P < 0.001, 95% CI = [0.411, 0.579]); loss aversion exhibited a significant partial mediating effect between MRI anxiety and treatment adherence (β = -0.142, SE = 0.047, 95% CI = [-0.233, -0.049]); and information avoidance also showed a significant partial mediating effect between MRI anxiety and treatment adherence (β = -0.132, SE = 0.032, 95% CI = [-0.203, -0.079]). Overall, loss aversion and information avoidance demonstrated a significant chain mediating effect in the relationship between MRI anxiety and treatment adherence (β = -0.178, SE = 0.030, 95% CI = [-0.241, -0.123]). The path coefficients are illustrated in **Figure 4**, thereby validating Hypothesis H4.

## 4 Discussion

### 4.1 The negative impact of MRI anxiety on treatment adherence

This study confirmed that MRI anxiety is negatively associated with patients’ treatment adherence. Specifically, MRI, as a non-invasive diagnostic tool, is associated with heightened emotions and cognition, which may correlate with increased anxiety and stress during the examination process (70), potentially relating to lower treatment adherence. This finding demonstrates that MRI anxiety is not only a subjective experience but also an objectively significant psychological phenomenon with clinical relevance. It expands our understanding of the sources of anxiety in healthcare settings. Traditional views have posited that medical anxiety primarily stems from the pain or side effects associated with treatment (71, 72). However, this study reveals that diagnostic tools themselves can become important psychological factors influencing treatment outcomes. Furthermore, certain objective factors within the healthcare environment may influence health behaviors by eliciting emotional responses. Therefore, a deeper understanding and intervention of MRI anxiety could enhance patients’ treatment adherence, ultimately improving overall therapeutic effects.

### 4.2 The mediating role of loss aversion

This study identified a mediating role of loss aversion between MRI anxiety and treatment adherence. These findings suggest that loss aversion, as a core psychological mechanism, amplifies patients’ perceived risks associated with MRI examinations, thereby intensifying their anxiety and subsequently reducing treatment adherence. This extends the application of loss aversion theory beyond its traditional focus on economic decision-making to the realm of emotional regulation in healthcare contexts. Furthermore, this discovery provides new empirical support for the health belief model, emphasizing that individuals’ perceptions of health threats and beliefs about treatment efficacy are critical factors influencing health behaviors (73). By elucidating the mediating pathway of loss aversion, this study reveals the psychological mechanisms underlying patients’ risk assessment processes during MRI examinations. This also opens new research directions inpatient psychological interventions, demonstrating that core concepts from economic decision-making theory hold significant explanatory power in medical psychology.

### 4.3 The mediating role of information avoidance

This study revealed the mediating role of information avoidance in the relationship between MRI anxiety and treatment adherence, providing a key cognitive mechanism to explain the behavioral withdrawal of high-anxiety patients. When faced with MRI examinations, patients tend to avoid relevant information, and this information processing strategy exacerbates their fear of the procedure, ultimately reducing treatment adherence. Based on the cognitive-emotional stress model (74), chronic stressors such as MRI anxiety can deplete limited cognitive resources, prompting individuals to adopt information avoidance strategies to alleviate cognitive overload and emotional discomfort. While this active cognitive avoidance may provide short-term emotional relief, it hinders comprehensive evaluation of risks and benefits and the development of coping strategies, ultimately weakening adherence. Thus, this study proposes a new pathway of fear-induced cognitive resource depletion and information avoidance leading to behavioral inhibition. This enhances the explanatory dimension of self-regulation theory in the context of health threat responses, highlighting the adaptive strategies individuals employ to maintain psychological stability under high emotional load.

This study revealed the mediating role of information avoidance in the relationship between MRI anxiety and treatment adherence, explaining behavioral withdrawal in anxious patients. Patients avoid MRI-related information (e.g., “I would rather not know the results of the MRI examination”), exacerbating fear and reducing adherence. This differs from loss aversion (e.g., “I would rather not think about MRI results because not knowing helps me maintain hope”), which emphasizes loss sensitivity. Per the cognitive-emotional stress model (74), MRI anxiety depletes resources, prompting avoidance for relief, but hindering risk evaluation. SEM results show distinct mediations (information avoidance: β = -0.142, p < 0.001; loss aversion: β = -0.159, p < 0.001; chain: β = -0.183, p < 0.001), confirming complementary roles: information avoidance as cognitive filtering, loss aversion as emotional bias. This enriches self-regulation theory in health threats.

### 4.4 Demographic information

The significant gender disparity in loss aversion highlights critical psychosocial dimensions of MRI anxiety. Females exhibited heightened sensitivity to potential health-related losses, aligning with broader literature on gender-specific risk perception in medical contexts. This may stem from sociocultural factors where women often assume primary health decision-making roles, amplifying anticipatory anxiety about diagnostic outcomes. Importantly, while gender influenced loss aversion, it did not significantly affect MRI anxiety itself, suggesting loss aversion operates as a distinct cognitive mediator rather than a direct correlate of baseline anxiety. These findings necessitate gender-tailored interventions, such as cognitive reframing techniques focused on loss-gain balance for female patients during pre-MRI counseling.

Age stratification revealed escalating loss aversion in older cohorts, reflecting cumulative health vulnerability. Older adults’ intensified focus on potential losses likely interfaces with comorbid health burdens and mortality salience, exacerbating MRI-related threat appraisal. Conversely, the absence of age effects on MRI anxiety or treatment adherence implies that while aging amplifies loss sensitivity, this manifests selectively rather than globally across psychological domains. Clinically, this underscores the need for age-specific communication strategies that address long-term health preservation benefits to counterbalance loss-focused cognitions in elderly populations.

Monthly income exerted a robust influence on MRI anxiety, loss aversion, and information avoidance. Paradoxically, both extremes of income exhibited elevated distress. Low-income groups face tangible stressors (e.g., out-of-pocket costs), while high-income patients may experience “hyper-vigilance” from heightened health literacy. This economic duality necessitates tiered interventions: financial counseling and sliding-scale fees for resource-constrained patients, and value-based framing emphasizing diagnostic efficacy for affluent subgroups to mitigate catastrophic thinking.

Examination location generated striking variations: abdominal MRI patients reported maximal anxiety, loss aversion, and information avoidance, whereas head/brain scans elicited the lowest scores. This anatomical gradient likely reflects visceral salience (e.g., abdominal organs symbolizing core vitality) and procedural discomfort (e.g., breath-holding requirements). The inverse pattern for treatment adherence further confirms that region-specific threats directly compromise compliance. These data argue for location-adjusted preparatory protocols, such as enhanced sensory mapping for abdominal scans to reduce unpredictability-driven avoidance.

First-time MRI recipients showed marked loss aversion and information avoidance. Surprisingly, MRI anxiety itself remained stable across exposure frequency, indicating that while procedural familiarity reduces cognitive avoidance mechanisms, visceral anxiety persists. This dissociation suggests that experiential interventions (e.g., virtual reality MRI simulators) should target informational coping rather than expecting anxiety extinction. The rise in treatment adherence with repeated scans further validates exposure-based desensitization for adherence optimization.

The absence of marital status effects contradicts theories linking social support to medical coping. Similarly, diagnostic indications (e.g., cancer vs. musculoskeletal) showed no psychological differentiation, suggesting MRI’s procedural characteristics dominate over condition-specific meanings. This reinforces MRI anxiety as a process-centric phenomenon requiring sensory-focused interventions rather than condition-specific counseling.

### 4.5 Practical implications

Based on the core finding that MRI anxiety significantly reduces patients’ treatment adherence, clinical practice urgently requires systemic innovations in screening and early intervention strategies. Traditional nursing protocols often underestimate or overlook the standardized assessment of pre-MRI anxiety, resulting in the failure to identify high-risk patients in a timely manner. This study confirms the direct negative effect of anxiety on adherence, strongly supporting the integration of efficient, standardized anxiety screening tools during the initial appointment or pre-screening phase. These tools should facilitate a tiered intervention approach based on the level of anxiety. For example, mild anxiety can be alleviated through enhanced informational support, while moderate-to-severe anxiety may require multidisciplinary collaboration, including cognitive behavioral therapy and, if necessary, the cautious use of short-term anxiolytic medications. Most importantly, these interventions should be implemented through optimized patient education and communication strategies, such as providing detailed examination information and emotional support to help patients manage their anxiety rationally. Such interventions not only improve treatment adherence but also enhance overall medical outcomes. By implementing these measures, healthcare teams can better assist patients in overcoming MRI anxiety, ensuring their active participation in treatment and achieving optimal therapeutic effects.

The critical mediating role of loss aversion in the relationship between MRI anxiety and treatment adherence provides new perspectives for designing precise interventions based on behavioral economics principles. Patients often amplify their fear by focusing excessively on potential losses, leading to avoidance of necessary examinations. This suggests that healthcare professionals should address patients’ loss sensitivity during communication. This can be achieved by emphasizing the efficiency and accuracy of the examination, reducing unnecessary focus on potential losses, and helping patients understand the necessity of the procedure from a positive perspective. Such psychological intervention strategies can reduce Patients’ loss aversion, lower MRI anxiety, increase treatment adherence, and ultimately improve therapeutic outcomes. This also implies that clinical staff should use structured decision aids to visually present the long-term benefits of timely examinations compared to the catastrophic consequences of delays. Training for healthcare professionals should focus on identifying and addressing loss-averse psychology, guiding patients to cognitive restructuring, and redefining MRI as an active, defensive investment in health.

The mediating role of information avoidance in the relationship between MRI anxiety and treatment adherence underscores the need for healthcare teams to adopt more transparent and moderate approaches to information disclosure. This not only helps patients acquire necessary knowledge but also reduces unnecessary concerns and anxiety. By optimizing information transmission strategies, such as using visual aids and simple, clear language, healthcare teams can effectively reduce patients’ information avoidance behaviors, alleviate MRI anxiety, and ultimately improve treatment adherence and overall therapeutic outcomes. This patient-centered approach to information disclosure fosters positive doctor-patient relationships, promoting the success of treatment.

### 4.6 Limitations and future research directions

Although this study provides valuable insights into the impact of MRI anxiety on treatment adherence and its underlying mechanisms, several limitations must be acknowledged. First, the use of convenience sampling, a non-probability method, may limit the representativeness of the sample (75). Participants were selected based on their availability in the Radiology Department of a tertiary hospital in Sichuan Province, without randomization, which may introduce selection bias and restrict the generalizability of findings to other regions, cultural backgrounds, or healthcare settings. The term “randomized convenience sampling” was avoided in this study to clarify that no probabilistic selection was employed, ensuring alignment with statistical terminology. Despite the robust sample size of 495 participants, which exceeded the minimum requirement calculated via G*Power (n = 119), the single-center design further constrains external validity. Factors such as medical resources in different regions, patient education level and cultural concepts may affect the relationship between MRI anxiety and treatment compliance. Therefore, future research can use multi-center, large-sample random sampling to carry out research in a wider range of regions and populations to enhance the universality of the conclusions.

Second, this study only performed a one-time data collection at the time of the MRI examination and lacked longitudinal follow-up of the patients before and after the examination. Treatment compliance is a dynamic process. Patients’ anxiety level and expectations before the examination may affect their treatment decisions and compliance behavior after the examination, and one-time measurement is difficult to capture this dynamic change. Future studies can design longitudinal research protocols to collect patients’ data at multiple time points before, during and after the examination, and deeply analyze the specific mechanism of MRI anxiety on treatment compliance at different stages, so as to provide a basis for clinical development of more targeted and staged interventions.

Third, the exclusive use of self-reported scales introduces potential measurement biases, such as social desirability bias, where patients may select responses aligned with perceived social norms, thus affecting data authenticity. Additionally, discrepancies may exist between self-reported anxiety levels and actual behaviors, as some patients reporting high anxiety may not exhibit reduced adherence in practice. To address this limitation, future research should triangulate self-reported data with objective behavioral and physiological measures to enhance validity. For instance, behavioral data, such as rates of missed MRI appointments, premature scan terminations, or non-compliance with radiographer instructions, can be extracted from medical records or radiology department logs to objectively assess treatment adherence (16). Similarly, physiological indicators, such as heart rate variability (HRV) or cortisol levels, can provide objective measures of anxiety during MRI examinations. HRV, in particular, is a non-invasive marker of autonomic nervous system activity and has been used to assess stress responses in medical imaging contexts (76). Wearable devices could be integrated into clinical settings to monitor HRV in real-time during MRI scans, offering a robust complement to self-reported anxiety measures. Combining these objective measures with self-reported data would provide a more comprehensive understanding of the interplay between MRI anxiety, loss aversion, information avoidance, and treatment adherence, mitigating the limitations of subjective reporting. Moreover, incorporating objective measures aligns with the cognitive-emotional stress model, which posits that chronic stressors deplete cognitive resources, influencing behavioral outcomes (74).

The study included participants with minor language, cognitive, or psychiatric impairments while ensuring that they were able to provide informed consent and complete the questionnaire, which may have led to variability in response. Although clinical assessment and standardized tools such as the MMSE rule out severe impairment, the presence of mild impairment may affect the accuracy of self-reported data, especially for scales assessing complex psychological constructs such as loss aversion and information avoidance. This variability may affect the reliability of responses, as patients with minor impairments may interpret or respond differently to questionnaire items. Future studies should adopt stricter inclusion criteria or employ standardized assessment tools to quantify the degree of impairment and ensure a homogeneous study population.

At the same time, although the model constructed in this study revealed the mediating role of loss aversion and information avoidance in the relationship between MRI anxiety and treatment adherence, the exploration of other potential influencing factors is limited. For example, factors such as the patient’s personality characteristics (e.g., neuroticism, extraversion, etc.), social support system, previous medical experience, and degree of trust in the health care system may also play an important role. Future research can further expand the model to include more potential moderating variables and mediating variables, and further explore the complex mechanism of MRI anxiety affecting treatment adherence, so as to provide more comprehensive theoretical support for optimizing clinical intervention strategies.

## Data Availability

The raw data supporting the conclusions of this article will be made available by the authors, without undue reservation.
